# Butyrate and Propionate Protect against Diet-Induced Obesity and Regulate Gut Hormones via Free Fatty Acid Receptor 3-Independent Mechanisms

**DOI:** 10.1371/journal.pone.0035240

**Published:** 2012-04-10

**Authors:** Hua V. Lin, Andrea Frassetto, Edward J. Kowalik Jr, Andrea R. Nawrocki, Mofei M. Lu, Jennifer R. Kosinski, James A. Hubert, Daphne Szeto, Xiaorui Yao, Gail Forrest, Donald J. Marsh

**Affiliations:** 1 Diabetes and In Vivo Pharmacology, Merck Research Laboratories, Rahway, New Jersey, United States of America; 2 Safety Assessment, Merck Research Laboratories, West Point, Pennsylvania, United States of America; University College Dublin, Ireland

## Abstract

Short-chain fatty acids (SCFAs), primarily acetate, propionate, and butyrate, are metabolites formed by gut microbiota from complex dietary carbohydrates. Butyrate and acetate were reported to protect against diet-induced obesity without causing hypophagia, while propionate was shown to reduce food intake. However, the underlying mechanisms for these effects are unclear. It was suggested that SCFAs may regulate gut hormones via their endogenous receptors Free fatty acid receptors 2 (FFAR2) and 3 (FFAR3), but direct evidence is lacking. We examined the effects of SCFA administration in mice, and show that butyrate, propionate, and acetate all protected against diet-induced obesity and insulin resistance. Butyrate and propionate, but not acetate, induce gut hormones and reduce food intake. As FFAR3 is the common receptor activated by butyrate and propionate, we examined these effects in FFAR3-deficient mice. The effects of butyrate and propionate on body weight and food intake are independent of FFAR3. In addition, FFAR3 plays a minor role in butyrate stimulation of Glucagon-like peptide-1, and is not required for butyrate- and propionate-dependent induction of Glucose-dependent insulinotropic peptide. Finally, FFAR3-deficient mice show normal body weight and glucose homeostasis. Stimulation of gut hormones and food intake inhibition by butyrate and propionate may represent a novel mechanism by which gut microbiota regulates host metabolism. These effects are largely intact in FFAR3-deficient mice, indicating additional mediators are required for these beneficial effects.

## Introduction

Short-chain fatty acids (SCFAs) are produced by microbiota in the colon and the distal small intestine from resistant starch, dietary fiber, and other low-digestible polysaccharides in a fermentation process [1]. Acetate, propionate, and butyrate are the predominant SCFAs in the gut lumen in humans and rodents, and are present at high mM levels [2]. Once produced, SCFAs are readily absorbed by colonocytes. Butyrate is largely utilized by the colonic epithelium as an energy source, and propionate is primarily utilized by the liver, whereas a significant amount of acetate enters systemic circulation and reaches peripheral tissues. In addition to acting as energy sources, SCFAs are also signaling molecules. The G protein-coupled receptors Free fatty acid receptor 2 (FFAR2, GPR43) and FFAR3 (GPR41) have been identified as endogenous receptors for SCFAs. Acetate preferentially activates FFAR2 in vitro; propionate displays similar agonism on FFAR2 and FFAR3; and butyrate preferentially activates FFAR3 [3], [4].

It is well established that supplementing resistant starch and dietary fibers in diet, which raises intestinal and circulating SCFAs, confers metabolic benefits in humans. In rodent models of genetic or diet-induced obesity, supplementation of butyrate in diet [5] and oral administration of acetate [6] was shown to suppress weight gain independent of food intake suppression. Activation of Adenosine 5'-monophosphate-activated protein kinase (AMPK) [5], [7] and increased mitochondrial function [5] were observed in these models, but only after chronic SCFA treatment when body weight was already significantly reduced compared to controls. Thus, the primary mechanism underlying the resistance to obesity remains obscure. Propionate was reported to inhibit food intake in humans [8], but the molecular mediators have not been identified. The SCFA receptors FFAR2 and FFAR3 are both expressed in the intestine and colocalize with a subset of enteroendocrine cells in the mucosal epithelium that express Peptide YY (PYY) [9], [10]. FFAR3 deficiency in mice was associated with an attenuated microbiota-induced increase in plasma PYY [11]. FFAR2 and FFAR3 are also expressed in other enteroendocrine subtypes [11]. PYY and other peptide hormones secreted by enteroendocrine cells, such as the incretins Glucagon-like peptide 1 (GLP-1) and Glucose-dependent insulinotropic polypeptide (GIP), are key modulators of energy homeostasis and glucose metabolism. Intracolonic and ileal infusions of mixed SCFAs were previously reported to increase PYY [12], [13], but the individual contribution of each SCFA was not determined. In addition, the effects of SCFAs on GLP-1 and other gut hormones have not been studied.

In this study, we examine the effects of SCFAs on body weight, glucose metabolism, and gut hormones in wild-type and *Ffar3* knockout mice. We show that butyrate and propionate suppress food intake, protect against high-fat diet-induced weight gain and glucose intolerance, and stimulate gut hormone secretion predominantly via FFAR3-independent mechanisms. We also show that FFAR3 is not required for normal body weight and glucose homeostasis.

## Results

### SCFAs Suppress Diet-induced Obesity through Distinct Mechanisms

**Figure 1:**
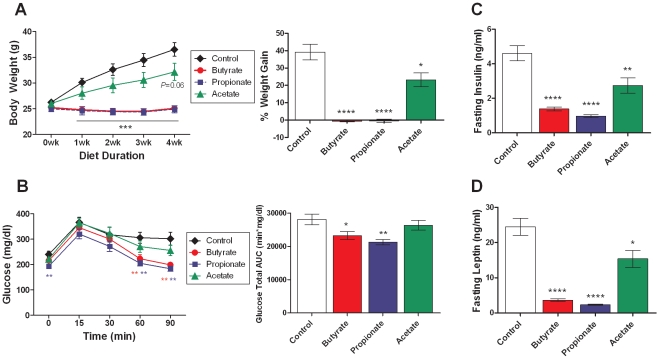
Effects of dietary SCFAs on body weight and glucose homeostasis. Three-month-old lean C57BL/6N mice were switched to HFD containing molarity-matched sodium salts of butyrate (5% w/w), propionate (4.3%), and acetate (3.7%) for four weeks. (A) Body weight was measured weekly, and four-week cumulative weight gain is expressed as a percentage of initial body weight. (B) Oral glucose tolerance test was performed in overnight fasted mice four weeks after diet switch. Blood glucose levels and total glucose area-under-the-curve (AUC) are shown. (C, D) Plasma levels of insulin and leptin were determined in overnight fasted mice four weeks after diet switch. Data are mean ± SEM. N=8. *P<0.05, **P<0.01, ***P<0.001, ****P<0.0001 vs. control HFD.

**Figure 2:**
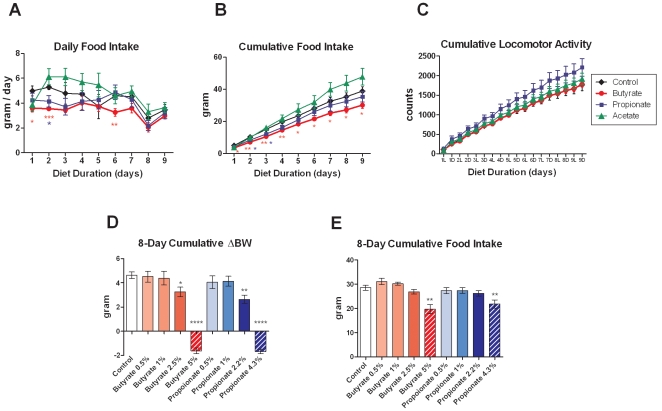
Effects of dietary SCFAs on food intake and locomotor activity. (A-C) Three-month-old lean C57BL/6N mice were switched to HFD containing sodium salts of butyrate (5%), propionate (4.3%), and acetate (3.7%) for nine days. Daily food intake, cumulative food intake, and cumulative locomotor activity are shown. L: light phase. D: dark phase. (D, E) Dose titration of sodium butyrate and sodium propionate in HFD was performed in three-month-old lean C57BL/6N mice. Eight-day cumulative body weight change and food intake are shown. Data are mean ± SEM. N=8. *P<0.05, **P<0.01, ***P<0.001, ****P<0.0001 vs. control diet.

To determine the effects of chronic SCFA treatment on the development of diet-induced obesity, three-month-old lean C57BL/6N mice were put on HFD supplemented with molarity-matched sodium salts of butyrate (5% w/w), propionate (4.3%), and acetate (3.7%) for four weeks. As expected, mice on control HFD gained weight steadily over time. Dietary supplementation of butyrate and propionate completely blocked HFD-induced weight gain, while acetate led to a 40% suppression of excess weight gain (Figure 1A). At the end of four weeks, propionate-fed mice showed reduced fasting glycemia, and both butyrate- and propionate-fed mice showed significantly improved oral glucose tolerance, while the acetate-fed group did not (Figure 1B). In addition, fasting insulin and leptin levels were significantly reduced by chronic supplementation of all three SCFAs (Figure 1C, D). These data are consistent with improvements in insulin sensitivity secondary to body weight reduction. In a separate cohort, food intake and locomotor activity were measured for nine days after mice were switched to control or SCFA-supplemented HFD. Butyrate significantly inhibited food intake on the first two days (Figure 2A), leading to a 22% reduction in nine-day cumulative food intake compared to control diet (Figure 2B). Propionate-fed mice also showed an initial reduction in food intake, resulting in a non-significant 9% reduction in nine-day cumulative food intake. In contrast, the acetate-fed group showed a non-significant 23% increase in cumulative food intake. Locomotor activity was not altered by butyrate or acetate, and tended to be increased by propionate feeding (Figure 2C). An eight-day dose titration study showed that the minimum efficacious dose for suppression of weight gain is 2.5% for butyrate and 2.2% for propionate (Figure 2D), and that for food intake inhibition is 5% and 4.3% for butyrate and propionate, respectively (Figure 2E). Acetate did not lead to a significant inhibition of weight gain during the first week (Figure 1A) and was not included in the dose titration study. Collectively, these data indicate that butyrate and propionate inhibit weight gain partially via suppressing food intake, while the inhibition of weight gain by acetate is independent of changes in food intake and locomotor activity, suggesting increased metabolic rate or reduced absorptive efficiency.

### Butyrate and Propionate Acutely Stimulate Gut Hormones

**Figure 3:**
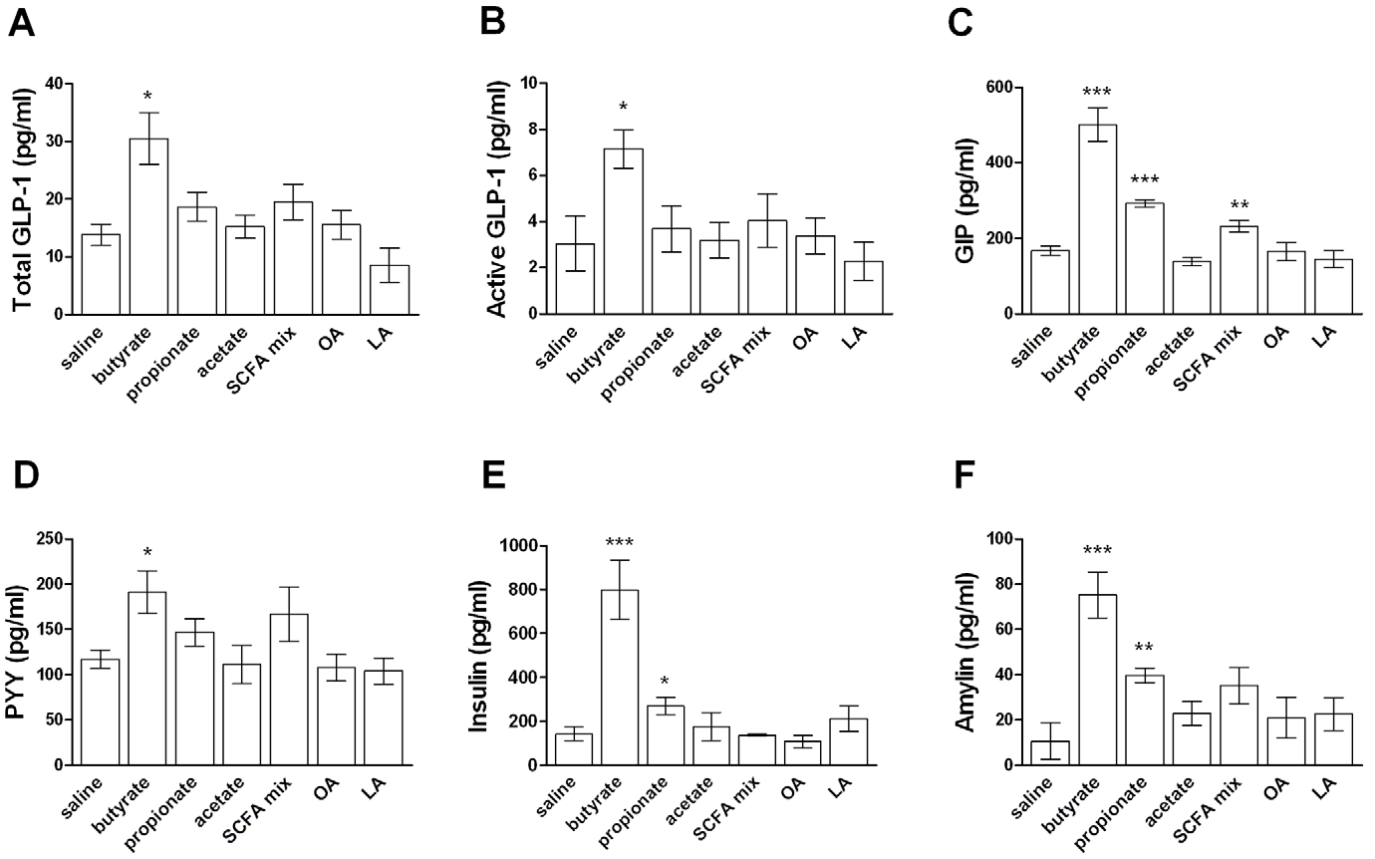
Effects of orally administered fatty acids on incretins and other hormones. (A-F) Three-month-old lean C57BL/6N mice were fasted overnight and orally dosed with saline, sodium butyrate, sodium propionate, sodium acetate, an SCFA admixture (65% sodium acetate, 20% sodium propionate, 15% sodium butyrate), octanoic acid (OA), or α-linolenic acid (LA), all at 400mg/kg body weight. Plasma levels of total GLP-1, active GLP-1, GIP, PYY, insulin, and amylin were measured 10 minutes after dosing. Intra-assay CV% was below 8.9% for all immunoassays. Data are mean ± SEM. N=8. *P<0.05, **P<0.01, ***P<0.001, ****P<0.0001 vs. saline. NS: not significant.

The colocalization of FFAR2 and FFAR3 with enteroendocrine cells in the intestine prompted us to examine the acute effects of SCFAs on gut peptides and other hormones. We selected the dose in the acute studies to match the amount of SCFAs consumed in a typical meal in the dietary supplementation experiment. Normal mice were reported to eat approximately 300mg HFD per meal ad libitum, or 10mg/g BW [14], [15]. Thus acetate, propionate, and butyrate (supplemented at 3.7 to 5%) were likely consumed at 370 to 500mg/kg per meal in the supplementation study. We therefore chose the dose of 400mg/kg for acute SCFA challenge. Plasma levels of the incretins GLP-1 and GIP were reported to peak at approximately ten minutes after an oral glucose challenge [16] and at 0.5 to 2 hours after an oral lipid challenge [17], respectively. We therefore examined gut hormones at ten minutes and one hour after oral SCFA challenge. Oral administration of sodium butyrate in mice significantly increased plasma levels of GLP-1 and GIP ten minutes after dosing (Figure 3A-C), and levels of both hormones normalized to baseline by sixty minutes post-dosing (data not shown). PYY also showed a moderate increase ten minutes after oral butyrate administration (Figure 3D). These changes were associated with elevated plasma insulin and amylin (Figure 3E, F) in butyrate-treated mice, consistent with stimulation of pancreatic β cells by incretins. Sodium propionate significantly increased GIP, insulin, and amylin, but not GLP-1 or PYY. An SCFA admixture mimicking the endogenous proportions present in colon (acetate 260mpk, propionate 80mpk, and butyrate 60mpk) also elicited a modest increase in GIP. In contrast, none of the hormones examined were significantly altered by sodium acetate dosed at either 400mg/kg (Figure 3) or 300mg/kg (molarity-matched to the sodium butyrate challenge, data not shown). In addition, the medium-chain fatty acid octanoic acid (OA) and the long-chain fatty acid α-linolenic acid (LA) also showed no significant effect. It's worth noting that the rank order of butyrate > propionate > acetate in the stimulation of anorexigenic peptides GLP-1, PYY, and amylin is consistent with their effects on food intake inhibition, with butyrate being the most potent (Figure 2A).

### Butyrate and Propionate Suppress Diet-induced Obesity in *Ffar3* Knockout Mice

**Figure 4:**
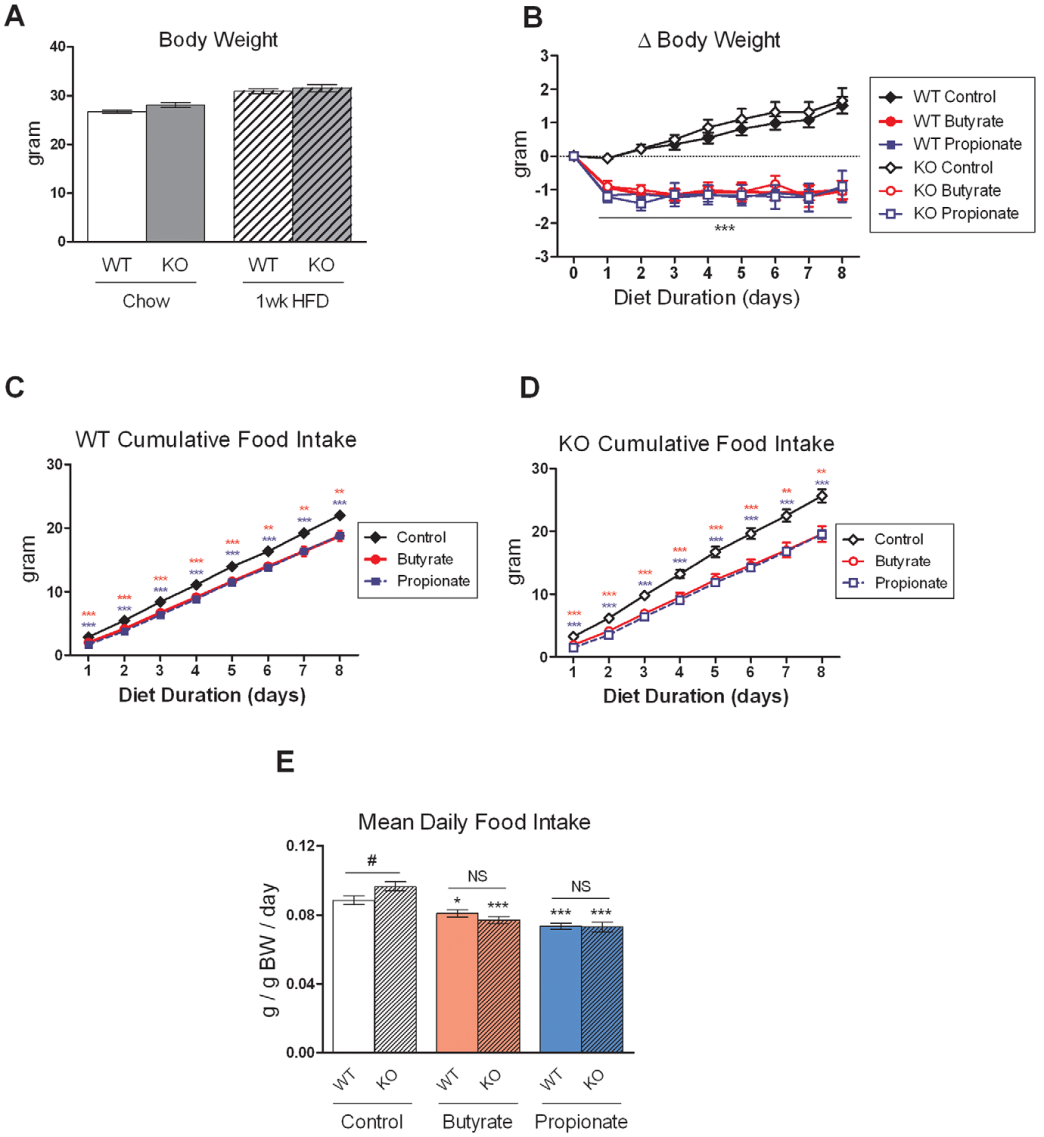
Effects of butyrate and propionate on energy homeostasis in *Ffar3* knockout mice. (A) Body weight of three-month-old *Ffar3* knockouts and wild-type littermates on standard chow diet and one week after switching to HFD. N=34–41. (B-D) After one week of HFD feeding, *Ffar3* knockouts and wild-type littermates were switched to HFD containing sodium butyrate (5%) or sodium propionate (4.3%) for eight days. Cumulative body weight change and daily food intake are shown. Data are mean ± SEM. N=8–13. *P<0.05, **P<0.01, ***P<0.001 vs. control diet. #P<0.05 vs. wild-type mice on control diet. NS: not significant.

Since butyrate and propionate, the SCFAs that preferentially activate FFAR3, are more effective than acetate in suppressing weight gain and stimulating gut hormones, we examined the contribution of FFAR3 to these effects. *Ffar3* knockout mice showed no significant difference in body weight compared to wild-type littermates on standard chow diet and after one week of HFD feeding (Figure 4A). The mice were then switched to HFD supplemented with butyrate and propionate for eight days. Both butyrate and propionate inhibited weight gain and food intake in *Ffar3* knockouts to the same extent as in wild-type mice (Figure 4B-D). Interestingly, although *Ffar3* knockouts on control HFD showed no difference in body weight compared to wild-type littermates, they showed a modest 9% increase in food intake (Figure 4E), consistent with reduced energy harvest efficiency from diet reported in a separate *Ffar3* knockout line [11].

### *Ffar3* Knockout Mice Show Normal GIP and Attenuated GLP-1 Induction by SCFAs

**Figure 5:**
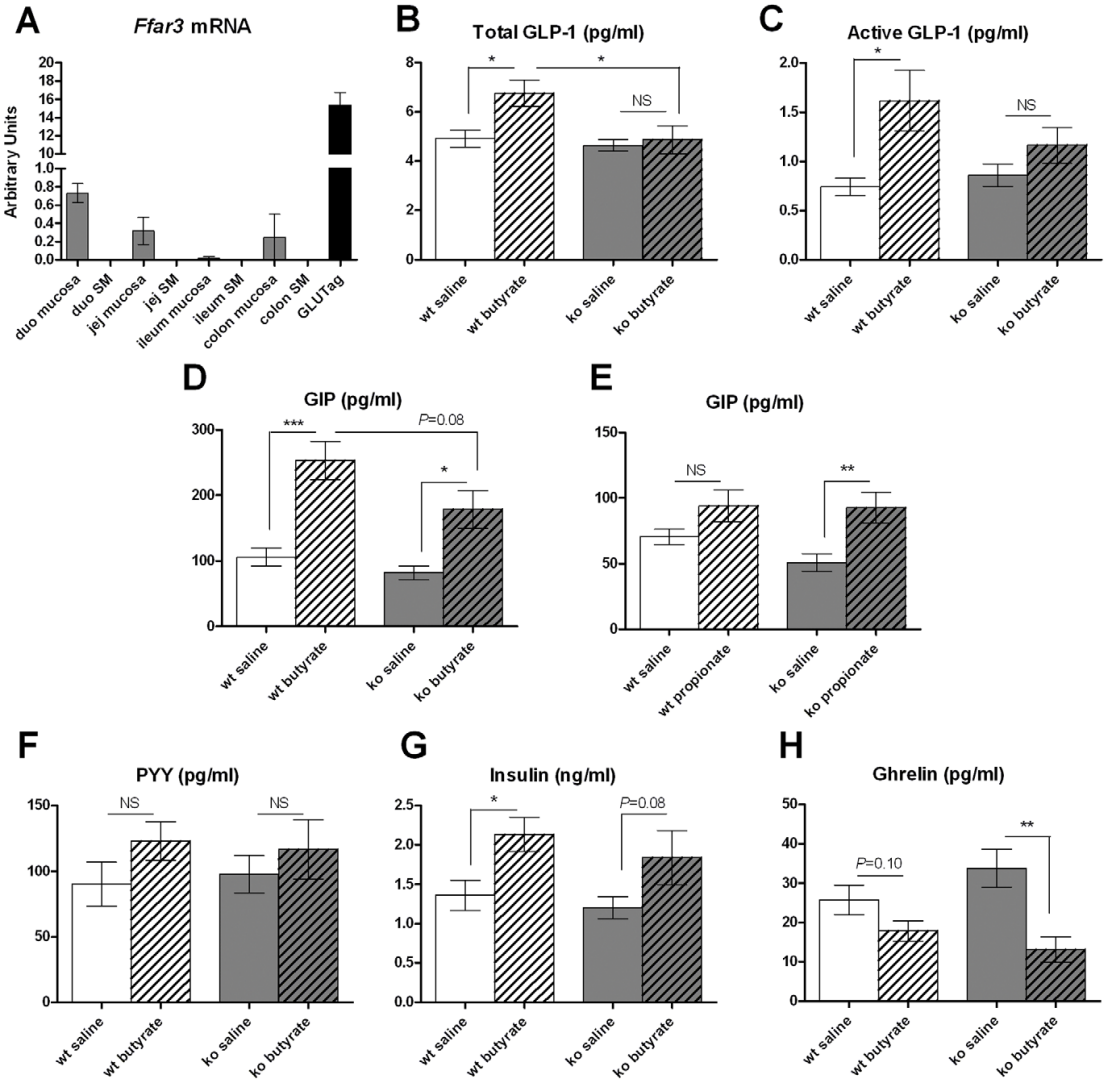
Effects of butyrate and propionate on gut hormones in *Ffar3* knockout mice. (A) *Ffar3* mRNA expression determined by quantitative RT-PCR in the mucosal and smooth muscle (SM) layers of different intestinal segments from lean C57BL/6N mice and in GLUTag cells. Data are normalized against *Rplp0* mRNA. N=3. (B-H) After four weeks of HFD feeding, five-month-old *Ffar3* knockout mice and wild-type littermates were fasted overnight and dosed with saline, sodium butyrate, or sodium propionate (400mg/kg). Plasma levels of total GLP-1, active GLP-1, GIP, PYY, insulin, and ghrelin were measured 10 minutes after dosing. Intra-assay CV% was below 7.6% for all immunoassays. N=8. Data are mean ± SEM. *P<0.05, **P<0.01, ***P<0.001, NS: not significant.

FFAR3 mRNA is expressed in the intestinal mucosa, and highly abundant in GLUTag cells, an immortalized murine L cell line, whereas it's undetectable in the intestinal smooth muscle layer (Figure 5A). This expression pattern is consistent with a potential role in peptide secretion from L, K, and other enteroendocrine cells. *Ffar3* knockouts showed normal fasting total and active GLP-1 levels, but butyrate induced total GLP-1 secretion was attenuated in the absence of FFAR3 (Figure 5B, C). GIP levels in *Ffar3* knockouts trended lower than wildtype controls under basal and butyrate-stimulated conditions, but the degree of stimulation by butyrate was similar between genotypes, and that by propionate showed a slight increase in knockouts (Figure 5D, E). Stimulation of PYY and insulin by butyrate was blunted in this cohort, likely due to exposure to HFD for four weeks, and showed no significant difference between genotypes (Figure 5F, G). These data suggest butyrate stimulation of GLP-1 secretion from L cells is partially mediated by FFAR3, whereas the stimulatory effects of butyrate and propionate on GIP secretion from K cells are FFAR3-independent. Interestingly, butyrate significantly reduced plasma ghrelin levels in *Ffar3* knockouts, while this effect did not reach statistical significance in control mice (Figure 5H).

### Normal Body Weight and Glucose Homeostasis in *Ffar3* knockout Mice

**Figure 6:**
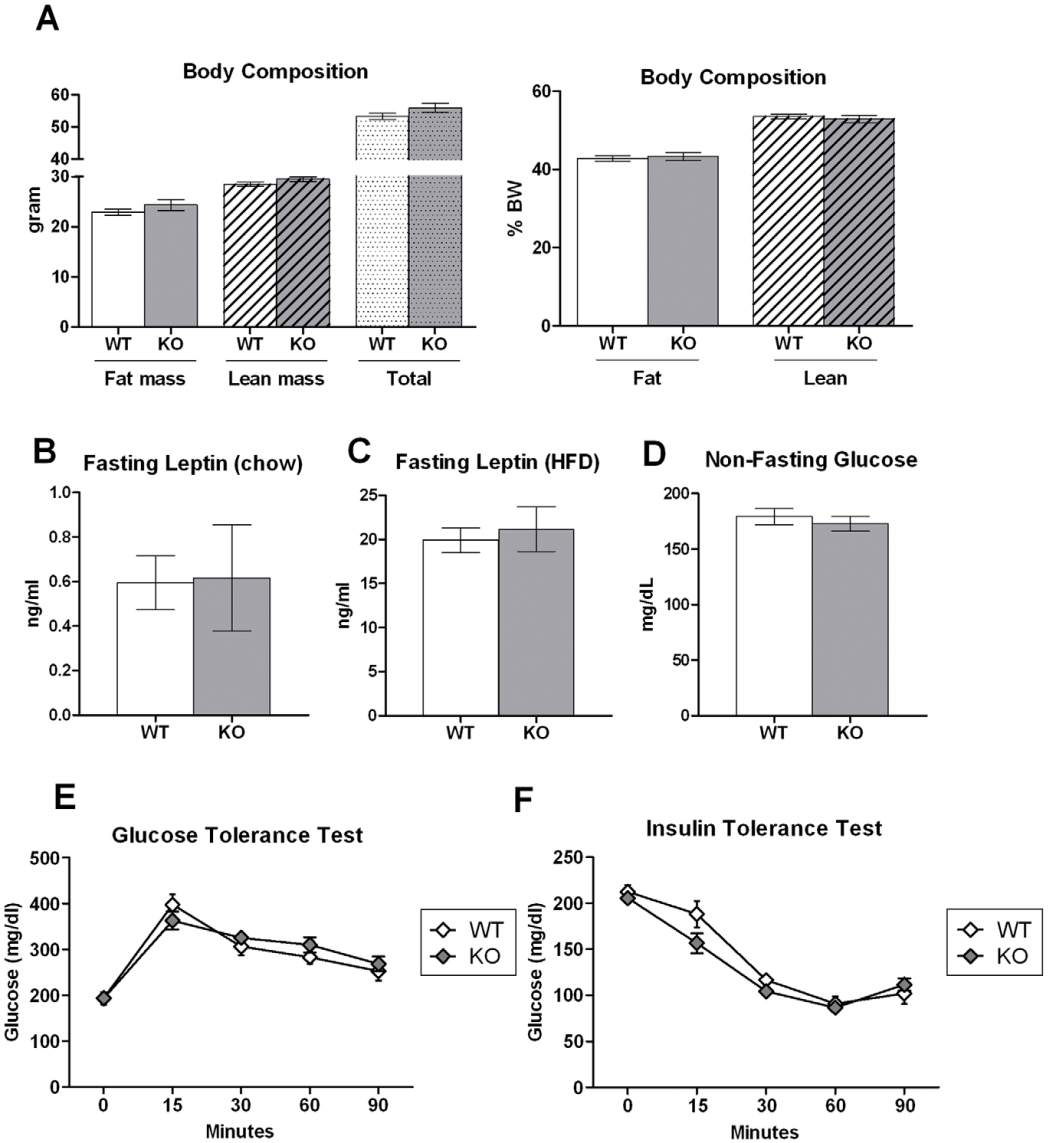
Normal body composition and glucose homeostasis in *Ffar3* knockout mice. (A) Body composition was determined by quantitative NMR in *Ffar3* knockouts and wild-type littermates after five months of HFD feeding. (B, C) Plasma leptin levels were determined in overnight fasted *Ffar3* knockouts and wild-type littermates maintained on standard chow diet or HFD. (D) Blood glucose was measured in ad libitum fed mice maintained on HFD three hours after the start of the light phase. (E) Oral glucose tolerance test after overnight fasting and (F) intraperitoneal insulin tolerance test after five-hour daytime fasting in *Ffar3* knockouts and wild-type littermates maintained on HFD. Data are mean ± SEM. N=8–14.

Both chow-fed (Figure 4A) and HFD-fed *Ffar3* knockout mice showed normal body weight, adiposity (Figure 6A), and plasma leptin levels (Figure 6B, C) compared to wild-type littermates. In addition, *Ffar3* knockout mice maintained on HFD showed normal glycemia (Figure 6D), oral glucose tolerance (Figure 6E), and insulin tolerance (Figure 6F). These data suggest that FFAR3 is dispensable for normal energy homeostasis and glucose metabolism.

## Discussion

The integral role of gut microbiota in the physiological regulation of host energy metabolism has attracted considerable attention. A number of studies have shown that obesity and metabolic disorders are associated with profound changes in gut microbiota [18]. However, mechanistic insights are lacking, and whether microbiota dysfunction plays a causal role in the pathogenesis of metabolic diseases is unclear. In particular, how microbiota-derived metabolites, such as SCFAs, interact with host nutrient sensing pathways to modulate energy metabolism is poorly understood. Although SCFAs have been postulated to regulate gut hormone secretion, in vivo evidence was scant, and the downstream signaling pathway was not characterized. In this study, we systematically examined the effects of each major SCFA naturally present in the colon–butyrate, propionate, and acetate–on energy metabolism and gut hormones. We found that all three SCFAs protected against diet-induced obesity, with butyrate and propionate being more effective than acetate. Butyrate and propionate regulate body weight at least partially by inhibiting food intake, consistent with their stimulatory effects on anorexigenic gut hormones. In contrast, acetate inhibited weight gain independent of food intake suppression and had no acute effect on gut hormones. Our finding on the hypophagic effect of butyrate differs from a previous report, which concluded butyrate supplementation led to hyperphagia [5]. As Gao et al. presented weekly food intake normalized to body weight, which was already significantly lower in butyrate-fed mice after the first week of diet switch, the acute hypophagic effect of butyrate may have been masked in that study. However, the current data cannot rule out potential impact on food intake due to altered palatability by butyrate and propionate supplementation. Although mice fed SCFA-supplemented diets did not display overt signs of malaise, additional studies will be required to formally address this possibility. In addition to effects on food intake, changes in energy expenditure likely also contribute to body weight regulation by SCFAs. Butyrate-treated mice showed an increased capacity for cold-induced adaptive thermogenesis [5]. Systemic administration of propionate acutely increases heart rate [19]. And acetate-treated rats were reported to have increased oxygen consumption [20]. Future studies will be needed to determine the contributions of these mechanisms to energy homeostasis in the chronic setting.

SCFA levels in the gut lumen were reported to reach high mM levels in the colon of human and pigs, but are much lower in jejunum and ileum [21]. In the current study, the doses of SCFAs chosen were in the high mM range, likely achieving supraphysiological levels in vivo, especially in the proximal intestine. Thus, the effects on gut hormones may not reflect physiological action of SCFAs, but suggest potential benefit of pharmacological SCFA treatment. Orally administered SCFAs can likely reach the proximal small intestine within 10 minutes–the early time point chosen for the acute studies–as gastric emptying of a liquid bolus in mice is very rapid and can exceed 80% within 15 minutes [22]. However, it’s unlikely that the oral SCFA bolus would reach the distal small intestine and colon within 10 minutes. Therefore, the effects on GLP-1 and GIP may reflect direct stimulation of enteroendocrine cells in the proximal small intestine, where both L cells and K cells can be found. On the other hand, L cells that express PYY are only present in the colon and distal small intestine, suggesting an indirect mode of action of butyrate on this gut hormone.

Butyrate- and propionate-dependent inhibition of food intake and weight gain was intact in *Ffar3* knockout mice, implicating other endogenous mediators in these effects. It's worth noting that *Ffar3* knockouts on control HFD showed modest hyperphagia but had normal body weight and adiposity. This is consistent with a previous report showing accelerated intestinal transit and increased fecal energy excretion in an independent *Ffar3* knockout line [11], suggesting FFAR3 is required for normal gut motility and nutrient absorption. The mechanisms responsible for increasing food intake and body weight normalization in the absence of FFAR3 are unknown. SCFAs were shown to regulate leptin secretion from the adipose tissue. However, the role of FFAR3 in mediating these effects remains controversial [23], [24]. We showed that *Ffar3* knockouts maintain normal plasma leptin levels, suggesting that leptin has no major role in normalizing energy homeostasis in *Ffar3* knockouts. *Ffar3* knockout mice were recently reported to have reduced resting heart rate and sympathetic activity [19]. However, this effect is expected to reduce energy expenditure and cannot explain the reduced feed efficiency of *Ffar3* knockouts.

Despite an intact anorectic response to butyrate, *Ffar3* knockouts showed an attenuation of butyrate-stimulated GLP-1 release. Fasting GLP-1 levels were normal in *Ffar3* knockouts. These data suggest FFAR3 plays a role in nutrient sensing in L cells but is not required for basal GLP-1 release. Conversely, *Ffar3* knockouts showed largely normal GIP stimulation by butyrate and propionate and an increased sensitivity to acute ghrelin suppression by butyrate. One potential explanation is that alterations in gastrointestinal motility in *Ffar3* knockouts may differentially affect delivery of orally administered SCFAs to enteroendocrine cell types along the proximodistal axis of the gut, contributing to the different sensitivities of various gut hormones to FFAR3 deficiency. Alternatively, FFAR3 may act as the primary butyrate sensor in L cells, while FFAR2 or additional SCFA sensors may play a more important role in other enteroendocrine cell types. Although butyrate displays poor agonism against FFAR2 in vitro, the local level present in the gut lumen after an oral administration may be sufficient to activate FFAR2 in the intestinal epithelium. In contrast to the adipose tissue, where FFAR2 expression tended to be reduced in FFAR3-deficient mice [25], we found FFAR2 mRNA expression to be normal in intestinal mucosa in *Ffar3* knockouts (data not shown). However, we cannot rule out the possibility that, in the absence of FFAR3, FFAR2 expression may be selectively up- or down-regulated in enteroendocrine cells, which only account for <5% of the intestinal epithelium. A recent report showed *Ffar2* knockout mice on HFD are protected from diet-induced obesity, most likely due to increases in energy expenditure and fecal energy output [26]. Future studies will be needed to determine the effects of SCFAs on body weight regulation and gut hormones in *Ffar2* knockouts.

Butyrate is a well-known histone deacetylase inhibitor and affects gene transcription [27]. Butyrate was also shown to regulate autophagy in colonocytes by acting as an energy source [28]. However, these mechanisms are unlikely to impact gut hormone release acutely on the scale of minutes. The niacin receptor GPR109A can also be activated by butyrate in the mM range and is expressed on the apical surface of colonic epithelium [29]. Its role in the regulation of gut hormone secretion and body weight remains to be determined.

In summary, the present findings demonstrate butyrate and propionate regulate gut hormone release, suppress food intake, and protect against diet-induced obesity. We also show that FFAR3 is required for maximal GLP-1 induction by butyrate, but is dispensable for butyrate- and propionate-dependent effects on body weight and GIP stimulation. As enteroendocrine nutrient-sensing and the incretin axis are subjects of intense interest in drug discovery for metabolic disorders [30], future studies to determine the signaling mechanisms responsible for SCFAs' beneficial effects may have a major impact on the development of novel therapies for diabetes and obesity.

## Materials and Methods

### Animals

C57BL/6N male mice were obtained from Taconic Farms (Germantown, NY), single-housed, and maintained in a 12h light/12h dark cycle. Diets used were either standard chow diet: Teklad 7012 (Harlan Teklad, Indiana, IN) or high-fat diet (HFD): D12492 (60% kcal from fat, Research Diets, New Brunswick, NJ). *Ffar3*-/- mice were obtained from Deltagen, Inc. (San Mateo, CA) and backcrossed to C57BL/6N for 6 generations. Knockouts and wild-type littermates were derived from heterozygote by heterozygote mating. Three primers were used to differentiate the mutant allele from the wild-type allele by PCR from tail DNA: 5'-GTGTAGGCAGTGTAGACAGCAATCT-3', 5'-GCAGAAGATGAAGGGCAGAAGCCAT-3', and 5'-GACGAGTTCTTCTGAGGGGATCGATC-3', which generate 609- and 357-bp amplicons from mutant and wild-type alleles, respectively. Male mice were used for all analyses. All animal procedures were approved by the Merck Research Laboratories Institutional Animal Care and Use Committee (Rahway, NJ) under permit numbers 10–058–07/11, 11–090–07/13, and 11–018–02/13.

### Acute Challenge and Dietary Supplementation Studies

Sodium butyrate, sodium propionate, sodium acetate, octanoic acid, and α-linolenic acid were obtained from Sigma-Aldrich (St. Louis, MO). For acute studies, mice were fasted overnight and dosed p.o. at 6ml/kg with compounds dissolved in saline. For dietary supplementation studies, sodium salts of SCFAs in solid form were thoroughly blended into high-fat diet using a food processor at 300–400rpm, formed into 50–60g balls, and used immediately. Control diet was similarly processed without the addition of SCFAs. In studies longer than 10 days, fresh diet was made and replenished weekly. For acute studies in *Ffar3* knockout mice and wildtype littermates, the same cohort that was used for the 8-day dietary supplementation study was returned to normal HFD for 3 weeks, then re-randomized and used for the acute butyrate/propionate challenge experiments due to limited animal availability. The magnitude of stimulation of active GLP-1 and GIP by butyrate was similar between wildtype mice on regular chow diet and those on 4wk HFD.

### Plasma Measurements

Blood was collected via cardiac puncture or submandibular bleeds into EDTA-tubes containing DPP4 inhibitor (Millipore, Billerica, MA) and protease inhibitor cocktail (Sigma-Aldrich). Total GLP-1 and active GLP-1 were measured using immunoassays from Meso Scale Discovery (Gaithersburg, MD). Total GIP, active PYY, insulin, amylin, leptin, and total ghrelin were measured using the Milliplex gut hormone panel (Millipore).

### Metabolic Analyses

Glucose tolerance test (3g/kg p.o.) was performed following an overnight fast in C57BL/6N mice that had been fed SCFA-supplemented HFD for four weeks and in five- to six-month-old *Ffar3*-/- and wild-type littermates that had been fed HFD for six weeks. Insulin tolerance test (0.75U/kg i.p.) was performed in six- to seven-month-old *Ffar3*-/- and wild-type littermates on HFD for ten weeks following a 5hr day-time food removal. Glucose was measured from tail blood with a OneTouch Ultra meter (LifeScan, Milpitas, CA). Body composition was determined with a Bruker minispec NMR (Bruker Optics, Billerica, CA) in eight-month-old *Ffar3*-/- and wild-type littermates that had been fed HFD for twenty weeks. Locomotor activity was measured using a comprehensive laboratory animal monitoring system (Columbus Instruments, Columbus, OH) as previously described [31].

### Statistical Analyses

Data were analyzed by Student's t test or one-way ANOVA with Bonferroni post test. P values less than 0.05 were considered significant.
